# Double-stranded RNA antiviral signature in early multiple sclerosis

**DOI:** 10.3389/fmicb.2025.1706133

**Published:** 2025-11-28

**Authors:** Vasileios Gouzouasis, Ioannis Sarrigeorgiou, Margaritis Tsifintaris, Nikos Markoglou, Urania Georgopoulou, Peggy Lymberi, Maria Anagnostouli, Lesley Probert, Antonis Giannakakis

**Affiliations:** 1 Department of Molecular Biology & Genetics, Democritus University of Thrace, Alexandroupolis, Greece; 2 Laboratory of Molecular Genetics, Department of Immunology, Hellenic Pasteur Institute, Athens, Greece; 3 Laboratory of Immunology, Department of Immunology, Hellenic Pasteur Institute, Athens, Greece; 4 Multiple Sclerosis and Demyelinating Diseases Unit, 1st Department of Neurology, School of Medicine, NKUA, Aeginition University Hospital, National and Kapodistrian University of Athens, Athens, Greece; 5 Laboratory of Molecular Virology, Department of Microbiology, Hellenic Pasteur Institute, Athens, Greece; 6 University Research Institute of Maternal and Child Health and Precision Medicine, National and Kapodistrian University of Athens, Athens, Greece

**Keywords:** double-stranded RNA, anti-dsRNA ELISA, multiple sclerosis, viral infection, Epstein–Barr virus (EBV), cytokines, cerebrospinal fluid, Poly(I:C)

## Abstract

**Introduction:**

Viruses, particularly Epstein–Barr virus (EBV), are strongly implicated in multiple sclerosis (MS) pathogenesis, yet reliable biomarkers of active viral replication remain limited. Double-stranded RNA (dsRNA) represents a hallmark of viral replication and may serve as a measurable indicator of viral infection.

**Methods:**

We developed a sandwich ELISA to quantify dsRNA levels in matched plasma and cerebrospinal fluid (CSF) samples from 70 treatment-naïve MS patients at first clinical onset and in plasma from 26 sex- and age-matched healthy controls. We additionally assessed plasma and CSF antiviral cytokine concentrations and designed an indirect ELISA to measure anti-dsRNA antibody levels using poly(I:C) as the target antigen.

**Results:**

Plasma dsRNA levels were significantly elevated in MS patients compared to controls and positively correlated with antiviral cytokines, including GM-CSF, IFN-λ1, IFN-λ2/3, IFN-γ, IFN-α2, and IL-12p70. CSF samples exhibited increased IP-10 and IL-8 levels, and the single patient with detectable CSF dsRNA showed among the highest concentrations of both cytokines. A subset of ten patients (14%) with serological evidence of atypical EBV reactivation (EBNA1 IgG^+^/IgM^+^) had higher plasma dsRNA and antiviral cytokine levels than the remaining patients. Anti-dsRNA IgM, but not IgG or IgA, correlated positively with plasma dsRNA in both MS patients and controls, yet anti-dsRNA IgM levels were significantly reduced in MS compared to controls.

**Discussion:**

Our findings identify increased plasma viral dsRNA coupled with reduced anti-dsRNA IgM antibody levels as potential biomarkers for a subpopulation of early MS patients and indicate a dysregulated anti-viral immune response.

## Graphical Abstract

**Figure:**
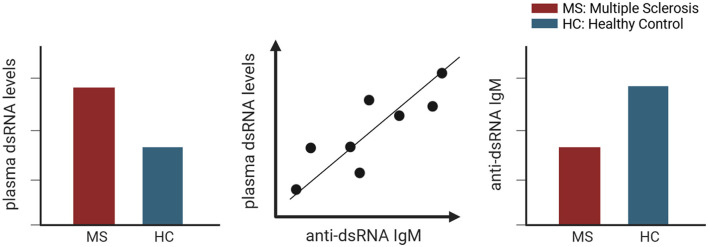

## 1 Introduction

Multiple sclerosis (MS) is a chronic inflammatory demyelinating disease of the central nervous system (CNS), with robust evidence for the involvement of viral infections in its pathogenesis (Bjornevik et al., 2022). Notably, several of the most effective and widely used MS therapies exert their effects either by stimulating antiviral immune responses (including interferon β) or depleting cells that harbor latent viral infections (including immune cells), underscoring a role of viruses in disease onset and progression (Bar-Or et al., 2020). Among the implicated pathogens, Epstein-Barr virus (EBV) has emerged as a key contributor (Bjornevik et al., 2022; Vietzen et al., 2023). EBV is a herpesvirus that typically causes infectious mononucleosis upon primary infection and subsequently establishes lifelong latency, primarily in B lymphocytes. Various environmental and immunological stressors can lead to EBV reactivation, which may in turn trigger or exacerbate autoimmune responses in MS (Indari et al., 2024).

A molecular hallmark of active viral infection is the formation of double-stranded RNA (dsRNA), which may derive from viral genomes or replication intermediates (Chen and Hur, 2022). dsRNA acts as a potent pathogen-associated molecular pattern (PAMP), activating pattern recognition receptors (PRRs) such as RIG-I, MDA5, and TLR3 (Chen and Hur, 2022; Jin et al., 2019; Uchikawa et al., 2016), and initiating downstream production of proinflammatory cytokines, which coordinate the body's response to infection (Chen and Hur, 2022). In line with this, previous research has focused on the identification of viral infection based on the measurement of dsRNA levels. For example, dsRNA immunofluorescence has been used for the identification of viral infection in respiratory human specimens and dsRNA immunohistochemistry to identify encephalitis in formalin-fixed, paraffin-embedded animal tissues (Kawang et al., 2021; de le Roi et al., 2025). In addition, a sandwich ELISA for the identification of dsRNA has been used in the process of mRNA vaccine development (Holland et al., 2024). Despite its diagnostic and mechanistic potential, dsRNA as a marker of dsRNA-derived inflammation has not been studied in the context of MS.

In addition to viral replication, dsRNA can arise endogenously within cells, through the epigenetic derepression of transposable elements, such as Alu elements, and defects in RNA metabolism, among others (Chen and Hur, 2022). In MS, there is widespread loss of A-to-I editing within Alu-dense regions (Tossberg et al., 2020). Because inosine destabilizes A:U base-pairing, loss of editing preserves dsRNA structure and potentiates recognition by RIG-I and TLR3, resulting in sustained inflammation (Tossberg et al., 2020). This leads to the formation of endogenous Alu-derived dsRNAs that induce type I interferon (IFN-I) responses (Heinrich et al., 2019) and provides a plausible mechanism through which self-derived nucleic acids can breach innate immune tolerance and perpetuate chronic inflammation (Ahmad et al., 2018). Although most dsRNA-sensing PRRs reside in the cytoplasm, it has been shown that dying cells release dsRNA in the extracellular space, which is up taken by recipient cells via specific receptors, such as SIDT2 and activates PRRs (Nellimarla and Mossman, 2014; Nguyen et al., 2017). Thus, extracellular dsRNA may originate from either viral or host sources.

In this study, we developed and optimized a sensitive sandwich ELISA for the detection of dsRNA levels in human samples. We used this ELISA to measure dsRNA levels in the plasma and cerebrospinal fluid (CSF) of therapy-naïve MS patients at the time of their first visit to the clinic, and the plasma of healthy control volunteers. To further explore the immunological response to dsRNA exposure, we also established an indirect ELISA to measure anti-dsRNA IgG, IgA, and IgM antibodies in plasma and CSF samples. We complemented data on dsRNA levels and anti-dsRNA antibody responses by measuring antiviral cytokines and anti-EBV antibody levels in plasma and CSF. Using these newly developed investigational tools, we provide novel insight into the presence of ongoing potential viral activity and its relationship with immune dysregulation in early-stage MS.

## 2 Materials and methods

### 2.1 Ethics statement

The study was conducted in accordance with the Declaration of Helsinki and approved by the Research Ethics Committees of the Democritus University of Thrace (Δ*ΠΘ*/EHΔE/25009/157, 20/12/2021) and Eginition Hospital, Medical School, National and Kapodistrian University of Athens (AΔA: 6IΨ646Ψ8N2-P9H, 05/07/2021). Written informed consent was obtained from all participants prior to inclusion in the study.

### 2.2 Sample collection and processing

The MS study cohort included 70 MS patients who presented at the outpatient clinic of the Multiple Sclerosis and Demyelinating Diseases Unit of the First Department of Neurology, Eginition Hospital, National and Kapodistrian University of Athens, Greece between May 2021 and March 2024, and received routine PB and CSF diagnostics as part of the full routine work-up for MS. Paired same-day plasma (*n* = 70) and CSF (*n* = 22) samples were collected from treatment-naïve MS patients at their first clinical visit and plasma samples from age-matched healthy controls (laboratory members and volunteers, *n* = 26), following informed consent. MS diagnosis was established by experienced neurologists based on internationally recognized criteria (Thompson et al., 2018). Inclusion criteria for MS patients required for this study: (i) absence of prior immunomodulatory treatment, including corticosteroids for at least 3 months before sampling, and (ii) diagnostic criteria consistent with MS.

Peripheral blood was collected in EDTA tubes and centrifuged at 1,200 × g for 15 min, acceleration: 7, deceleration: 3, at 23 °C to separate plasma from cellular components. Supernatants were aliquoted and stored at −80 °C until analysis. CSF samples were obtained via lumbar puncture, centrifuged at 1,200 × g for 10 min to remove cellular debris, and stored at −80 °C.

### 2.3 dsRNA detection ELISA

To detect dsRNA in biological fluids, we developed a sensitive sandwich ELISA using two primary anti-dsRNA monoclonal antibodies and one secondary antibody. For dsRNA capture, we employed the well-characterized mouse anti-dsRNA IgG2a (κ chain) monoclonal antibody J2 (Scicons, catalog #10010500; Nordic-MUbio), which binds sequence-independent dsRNA helices of ≥40 base pairs (Schönborn et al., 1991). Capsured dsRNA was detected with the mouse anti-dsRNA IgM (κ chain) monoclonal antibody K2 (Scicons, catalog #10030010; Nordic-MUbio), an isotype alternative to J2 that also binds dsRNA ≥40 base pairs and is recommended for sandwich ELISA (Schönborn et al., 1991). To visualize binding, we used a horseradish peroxidase (HRP)-conjugated goat F(ab')_2_ anti-mouse IgM μ-chain secondary antibody (Abcam, ab5930).

To optimize assay sensitivity, standard curves were generated based on optical density (OD) measurements at 450 nm. A pre-defined threshold of OD > 2.0 was set to define optimal signal intensity. Serial twofold dilutions were prepared, starting at 1:500 for J2 and 1:20 for K2. The optimal dilutions were determined at 1:1,000 for J2 (yielding an OD = 2.58, second dilution step) and 1:20 for K2 (OD = 2.91, first dilution step).

Both J2 and K2 antibodies detect a broad range of viral and synthetic dsRNA species. Assay calibration and standardization were performed using polyinosinic:polycytidylic acid [Poly(I:C)] (catalog #4287, Tocris Bioscience), a synthetic dsRNA analog. As a biological positive control, we employed hepatitis C virus (HCV, JFH-1 strain) RNA preparations, as described previously (Walker, 1984). To generate HCV dsRNA (HCV-T1 cut), 10 μg of HCV RNA were pre-incubated for 1 h at 37 °C with RNase T1 (10 Units), which selectively cleaves ssRNA without affecting dsRNA. The resulting dsRNA (1.7 μg, 17% of the total HCV RNA) was purified using RNA Clean-Up and Concentration Micro-Elute columns (Norgen) and quantified with Nanodrop.

Negative controls included thymus DNA and RNase III-digested Poly(I:C) or HCV RNA, to confirm the assay's specificity for intact dsRNA over DNA or single-stranded RNA (ssRNA). In addition to RNase III, RNase 1 and RNase T1 were used for the generation of positive and negative controls as follows. First, 100 μl Poly(I:C) 1 mg/ml (Tocris, 4287) were diluted in 10 ml PBS to obtain a concentration of 1 μg in 100 μl. 1 U of each RNase was used per reaction, and the corresponding reaction buffers were prepared according to the manufacturer's instructions. For RNase III reaction, 1 μl Ambion™ RNase III (1 U/μL, Invitrogen™, AM2290) were mixed with 9 μl RNase III buffer and 90 μl Poly(I:C) (0.9 μg) and incubated for 1 hr at 37 °C. For RNase T1 reaction, 1 μl RNase T1 (1,000 U/μL, Thermo Scientific™, EN0541) was first added in 1 ml RNase T1 buffer, from which 1 μl was incubated with 99 μl of Poly(I:C) (0.99 μg) for 30 min at 37 °C. For RNase 1 reaction, 1 μl RNase 1 (10 U/μL, Thermo Scientific™, EN0601) was added to 9 μl of RNase 1 buffer, from which 1 μl was incubated with 99 μl of Poly(I:C) (0.99 μg) for 30 min at 37 °C. When RNase treatment was performed on HCV RNA, 0.5 μg of either HCV, or HCV-T1 cut were used per reaction. All RNase digestions were carried out for an additional hour at room temperature (RT) during sample incubation.

High-binding 96-well ELISA plates (Nunc, MaxiSorp^®^) were coated with 10 μg/mL Protein A in PBS (100 μL per well) overnight at 4 °C with gentle shaking (250 rpm). Plates were washed three times with washing buffer (WB; PBS with 0.5% Tween 20), wells were coated with 100 μL of J2 antibody (1:1,000 in blocking buffer, BB; PBS with 0.5% BSA) and incubated overnight at 4 °C with gentle shaking (250 rpm). Plates were washed three times with WB and incubated with 100 μL diluted plasma samples (1:100 in PBS), or undiluted CSF samples. Plates were incubated at RT for 1 h, washed 3 times with WB, and incubated with 100 μl of K2 detection antibody (1:16,000 in BB) for 1 h at 4 °C with gentle shaking (250 rpm). Plates were washed three times with WB and incubated with HRP-conjugated secondary antibody (1:20,000 in BB) for 1 h at 4 °C. Plates were washed 4 times with WB and incubated with chromogenic substrate TMB (3,3′,5,5′-Tetramethylbenzidine) for 15 min at RT. The enzyme reaction was stopped with 1 M sulfuric acid, and the OD was measured at 450 nm using a TECAN photometer (TECAN Spark Control Magellan V2.2, Grödig/Salzburg, Austria). For MS patients, dsRNA levels were classified in relation to the distribution of reactivity values observed in healthy control samples. Specifically, sera from MS patients with values exceeding the upper standard deviation (SD) limit of healthy controls were defined as exhibiting high reactivity, those falling within the control SD range as moderate reactivity, and those below the lower SD limit as low reactivity.

### 2.4 Anti-dsRNA antibody detection ELISA

ELISA microplates (675061, Greiner Bio-One, Kremsmünster, Austria) were coated with Poly-L-lysine (0.01%) (P4832, Sigma) for 1 h at 37 °C. After extensive washing with PBS, plates were coated with capture dsRNA antigen [Poly(I:C)] (10 μg/mL in PBS 0.1 M, pH 6.8), overnight at 4 °C. Plates were washed four times with PBS, incubated with BB for 1 h at 37 °C and incubated with plasma samples (1:100 in BB containing 0.05% Tween 20) overnight at 4 °C. Plates were washed extensively with WB and incubated with alkaline phosphatase-conjugated secondary antibodies against human IgM (Jackson ImmunoResearch, West Grove, PA, cat #109-055-129), IgA (catalog #109-055-011), or IgG (catalog #109-055-088) at a final concentration of 0.1 μg/mL for 2 h at 37 °C. Plates were washed and incubated with chromogenic substrate 4-nitrophenyl phosphate disodium salt hexahydrate (pNPP; N2765, Sigma). The OD of the reaction product was measured at 405 nm with a 620 nm reference using a TECAN photometer (TECAN Spark Control, Magellan v2.2, Grödig/Salzburg, Austria). For interassay normalization, three positive (sera exhibiting high reactivity for dsRNA) and three negative (sera with low reactivity for dsRNA) controls were included on each plate, and interassay variability was maintained below 10%.

### 2.5 EBV ELISA

Commercially available ELISA kits were used to analyze plasma samples for biomarkers of EBV to determine EBV infection status (ELISA-VIDITEST assays; VIDIA). The EBV antibody panel comprised anti-EBNA1 IgG (cat #ODZ-001), anti-EBNA1 IgM (cat #ODZ-002), anti-EA(D) IgG (cat #ODZ-006), anti-VCA IgG (cat #ODZ-265), anti-VCA IgM (cat #ODZ-005), and anti-VCA IgA (cat #ODZ-096). Interpretation of the results was performed as shown in Table 1 and previously described (Gouzouasis et al., 2025; Christian Münz, 2015).

**Table 1:** Clinical interpretation of EBV results based on the ELISA kit manufacturer's instructions.

**Clinical interpretation of results**							
**VCA EBV**			**EA(D) EBV**		**EBNA1 EBV**		**Stages of EBV infection**
**IgG**	**IgM**	**IgA**	**IgG**	**IgM**	**IgG**	**IgM**	
–	–	–	–	–	–	–	Seronegative
–	+	+	–	±	–	+	Primoinfection (early phase)
+ Low avidity	+	+	±	±	–	+	Primoinfection
	+	-	±	±	–	+	
	-	+	±	±	–	–	
+ High avidity	+	-	±	–	+	–	Suspected reactivation
	-	+	±	–	+	–	
	-	-	++	–	+	–	
+ High avidity	–	–	–	–	+	–	Seropositivity without symptoms of active infection
+	±	±	±	±	+	+	Atypical reactivation

### 2.6 Antiviral cytokines

Cytokine profiling was performed to measure plasma levels of the following antiviral cytokines: IL-1β, IL-6, IL-8, IL-10, IL-12p70, IFN-α2, IFN-β, IFN-λ1, IFN-λ2/3, IFN-γ, TNF-α, IP-10, and GM-CSF. Measurements were carried out using a LEGENDplex™ multiplex assay (catalog #740390, BioLegend), and data acquisition was conducted on a BD FACSCalibur™ flow cytometer.

### 2.7 Statistical analysis

The D'Agostino-Pearson normality test was used to assess the normality of the data distribution for each analysis. For comparisons between two independent groups, the *t*-test was used for parametric data, while the Mann–Whitney U test was used for non-parametric data. For comparisons among more than two independent groups, the ANOVA test was used for parametric data, and the Kruskal–Wallis test was used for non-parametric data. Correlation between continuous variables was assessed using Pearson's correlation coefficient for parametric data and Spearman's rank correlation coefficient (rho) for non-parametric data. In all cases, the significance level was set at 5%. Tests were two-tailed, and a result was considered statistically significant if the estimated *p*-value was less than the significance level (p < 0.05). Statistical analysis and graph generation were performed using GraphPad Prism version 9.0.0 (GraphPad Software, San Diego, California, USA).

## 3 Results

### 3.1 Development of a sensitive and specific sandwich ELISA for quantification of total dsRNA in plasma and CSF

**Figure 1:**
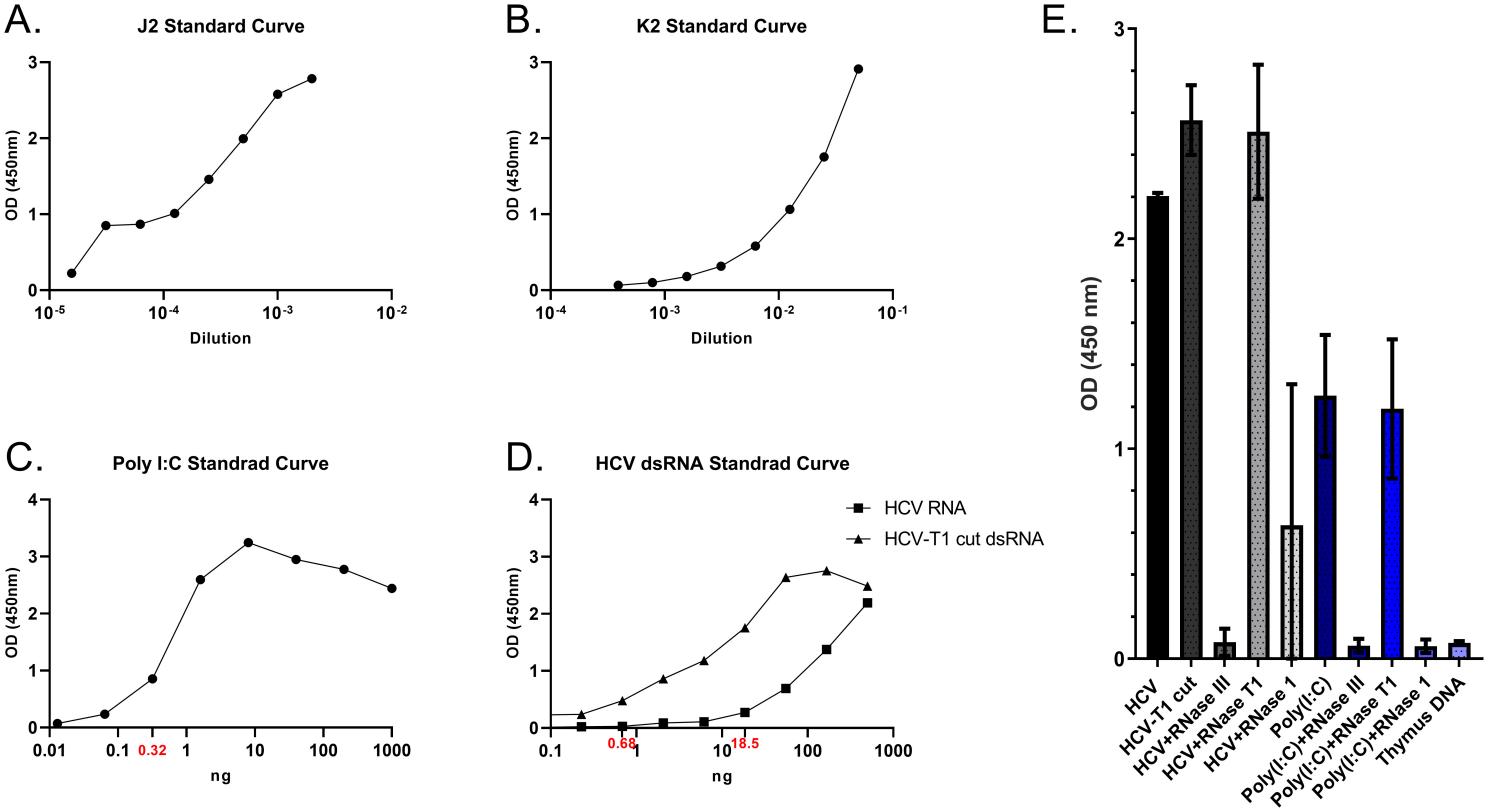
Sensitivity and specificity of the sandwich ELISA for the detection of dsRNA. **(A)**. J2 standard curve; J2 serial $\frac{1}{2}$ dilutions were used starting at 1/500 J2 diluted in BB **(B)**. K2 standard curve; K2 serial $\frac{1}{2}$ dilutions were used starting at 1/20 K2 diluted in BB. **(C)**. Poly(I:C) standard curve; Poly(I:C) serial _1/5_ dilutions were performed starting at 1 μg Poly(I:C) diluted in PBS. **(D)**. HCV standard curves; HCV serial $\frac{1}{4}$ dilutions were used starting at 0.5μg HCV RNA and HCV-T1 cut dsRNA. **(E)**. Controls for dsRNA detection; Absorbance at 450 nm reflects dsRNA signal under various experimental conditions, when 0.5 μg HCV, 0.5 μg HCV-T1 cut and 1 μg Poly(I:C) were added for each reaction in the ELISA plate. Error bars represent the standard deviation. Experiments were conducted in triplicate, and replicate values obtained from a single ELISA plate were selected for graphical representation in **(A–D)**.

To establish a specific and sensitive sandwich ELISA for dsRNA detection, we first standardized the individual assay components, including dsRNA capture and detection antibodies, positive and negative controls, and experimental conditions. Based on serial dilution experiments, a 1:1,000 dilution of the capture antibody J2 (Figure 1A) and a 1:20 dilution of the detection antibody K2 (Figure 1B) were selected as optimal. These concentrations provided high signal intensity while maintaining assay specificity for subsequent analyses. Further optimization was performed to address background noise and enhance assay specificity and sensitivity. Initially, a Goat Anti-Mouse IgM μ-chain HRP-conjugated secondary antibody (Goat Anti-Mouse IgM mu chain (HRP), catalog # ab97230; Abcam) was used for the detection of the sandwich ELISA but resulted in increased background signal. Subsequently, a Goat F(ab‘)_2_ Anti-Mouse IgM (μ-chain) HRP-conjugated antibody (catalog # ab5930, Abcam) was used, which gave no background signal. In addition, protein A pre-coating of the plate significantly enhanced the specific dsRNA detection signal by increasing the plate coating efficiency of J2 antibody and was included in all following experiments. By incorporating the use of a F(ab')_2_ secondary antibody and Protein A precoating, we significantly enhanced the assay's signal-to-noise ratio and minimized background signal compared to previously published protocols (Holland et al., 2024).

For the development of the sandwich ELISA, poly(I:C) was selected as a positive reference for a dsRNA species. A standard curve was generated by testing 1/5 serial dilutions of poly(I:C), starting at 1 μg (Figure 1C). A characteristic hook effect was observed where excessive concentrations of Poly(I:C) resulted in a reduction of signal intensity. To mitigate this problem, we explored usage of alternative positive reference samples, such as HCV RNA preparations (see methods), which demonstrated superior absorbance when measured at 450 nm. Two standard curves were generated: one for total HCV RNA, starting at 0.5 μg and serially diluted $\frac{1}{4}$, and one for an HCV dsRNA-enriched RNA (referred to as HCV-T1 cut), which had been previously treated with RNase T1, starting also at 0.5 μg dsRNA using $\frac{1}{4}$ dilutions (Figure 1D). This approach enabled the generation of a standard curve, allowing quantification of dsRNA with a lowest detection threshold of 0.32 ng of Poly(I:C), 0.68 ng of HCV-T1 cut, and 18,5 ng total HCV RNA. The observed difference between HCV-T1 cut and HCV RNA detection was expected, given that the HCV RNA input contained a mixture of ssRNA and dsRNA, a finding consistent with the high sensitivity of our technique. Accordingly, HCV-T1 cut, which provided a well-defined and specific signal, was selected as the standard for quantification, enabling accurate measurement of dsRNA levels in plasma samples. To our knowledge, this approach for quantifying dsRNA levels in biological fluids has not been previously reported.

Additional measures to validate the specificity of the assay included the introduction of a negative thymus dsDNA sample and three RNase treatments to create internal negative controls for HCV and poly(I:C) samples. RNase III, which selectively cleaves dsRNA, RNase T1, which selectively cleaves ssRNA and RNase 1, which mainly cleaves ssRNA but can also cut dsRNA when used in excess levels. Incubation of the positive controls, Poly(I:C), HCV RNA and HCV-T1 cut dsRNA, with any of the above 3 RNases, confirmed the assay's specificity for dsRNA detection (Figure 1E). RNase T1 did not cleave either HCV or Poly(I:C) dsRNA, while RNase III efficiently degraded both (> 1 Unit/μg sample), as expected. On the other hand, RNase 1 fully cleaved Poly(I:C) dsRNA (≥1 Unit/0.5 μg Poly(I:C)). In total, our technique demonstrated high specificity for dsRNA, without cross-reactivity with thymus dsDNA.

### 3.2 Plasma dsRNA levels are increased during the first clinical visit of MS compared to healthy individuals

**Figure 2:**
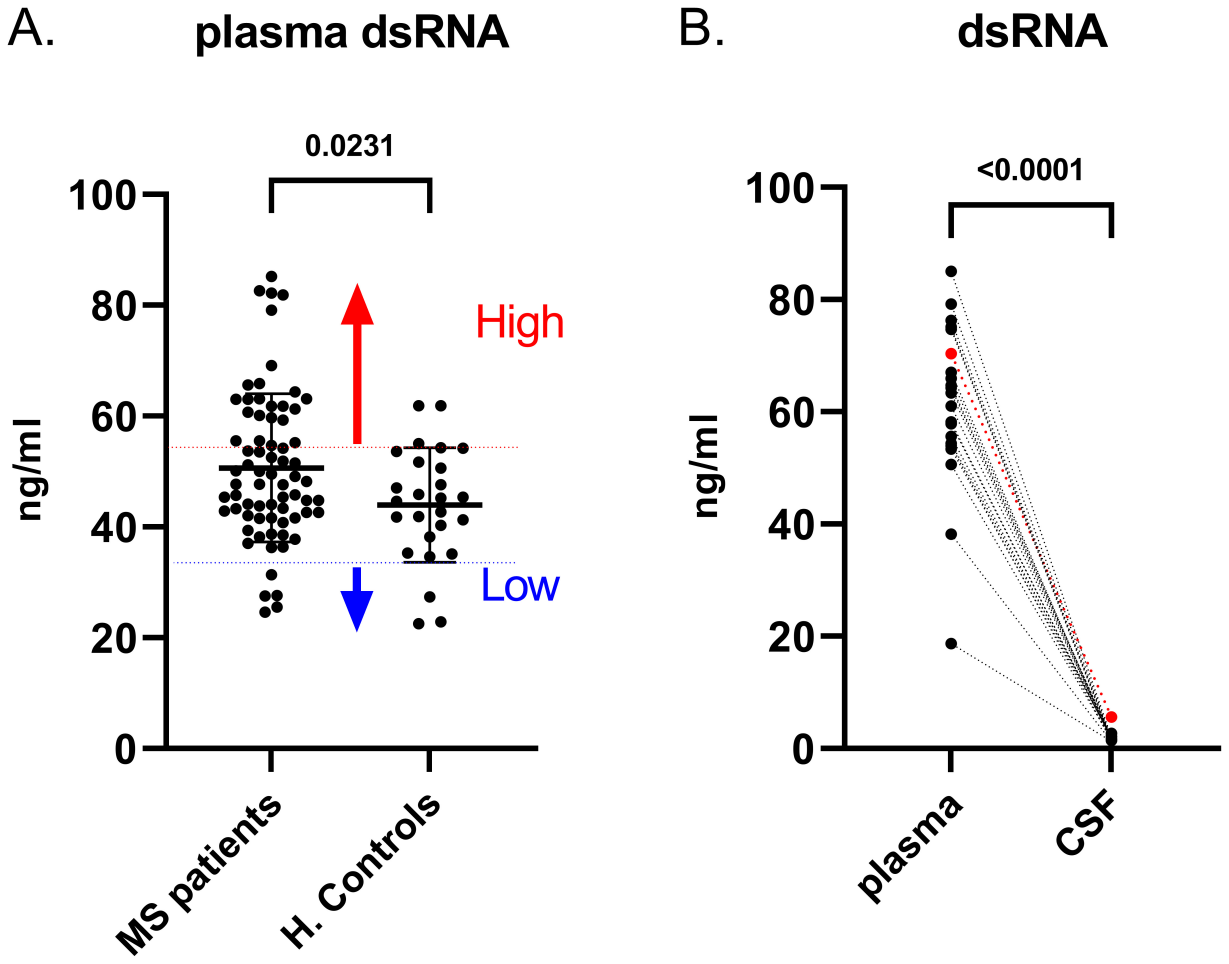
Increased Plasma Double-Stranded RNA (dsRNA) Levels During the First Clinical Visit of MS. **(A)**. Plasma dsRNA levels in MS patients (*n* = 70) compared to healthy controls (*n* = 26). **(B)**. Detection of dsRNA in plasma and CSF of MS patients (*n* = 22). CSF dsRNA levels were significantly lower than plasma levels. The highest OD value in the CSF represents Patient E (red).

To evaluate the application of the optimized sandwich ELISA to clinical samples, we assessed dsRNA levels in plasma and CSF of MS patients and in plasma of healthy controls. Plasma samples from MS patients consistently exhibited significantly higher dsRNA levels (*N* = 70; 51 ±13 ng/ml) compared to healthy controls (*N* = 26; 44 ±10 ng/ml) across three independent experiments, as determined by a Mann-Whitney U test (p = 0.0231) (Figure 2A). Among the MS plasma samples tested, 24 showed high dsRNA levels, 43 exhibited moderate levels, 3 had low levels. The raw data are presented in Supplementary Table 1.

Based on our analysis, all CSF samples (in MS patients and Healthy individuals) were negative for dsRNA except for one patient (named hereafter as Patient E) who consistently exhibited detectable dsRNA levels in the CSF among the other patients' samples, as measured by four independent experiments (Figure 2B). This patient also showed serological evidence of atypical EBV reactivation (concurrent anti-EBNA1 IgG and IgM positivity), together with anti-VCA IgM antibodies in the plasma and anti-EBNA1 IgG in CSF. The diagnostic work-up for this patient revealed clinically and radiologically active disease and the presence of oligoclonal bands in CSF. Further investigation in a larger cohort, including expanded viral screening, will be necessary to determine whether CSF dsRNA reflects viral persistence.

### 3.3 Plasma dsRNA levels positively correlate with levels of antiviral cytokines

**Figure 3:**
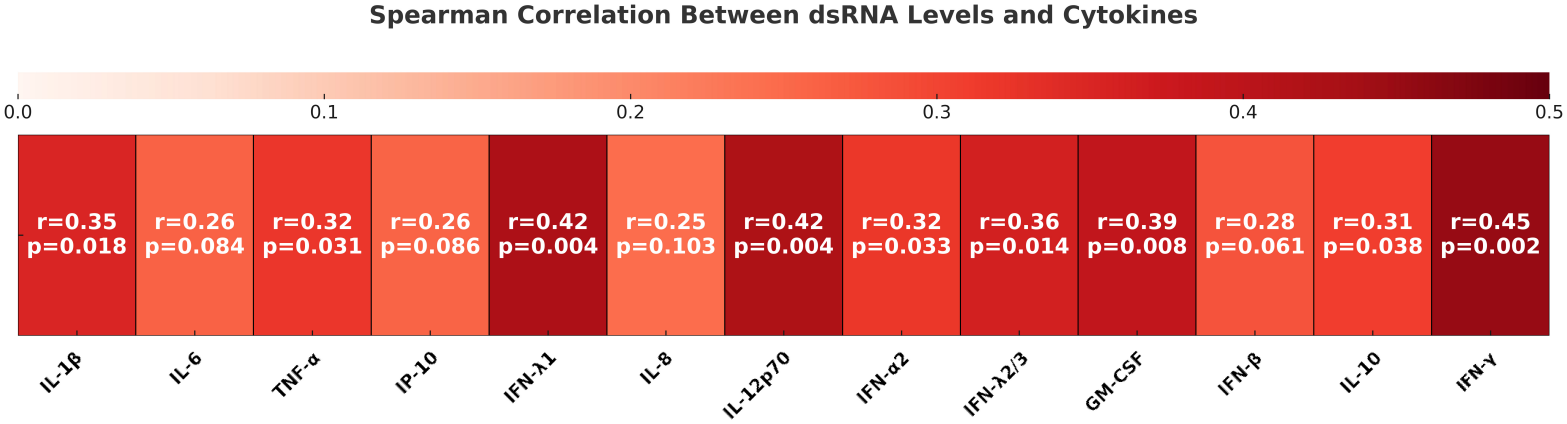
Heatmap analysis for Spearman correlation values between the levels of dsRNA and antiviral cytokines in plasma samples from MS patients. Spearman correlation coefficients (r) are shown and visualized using the color scale shown at the top, and the corresponding *p* values are indicated.

To assess immune activation status in our cohort, we quantified 13 chemokines/cytokines in the plasma and CSF of MS patients (Figure 3). Strikingly, several cytokines, including IFN-λ1, IFN-λ2,3, IFN-γ, IL-12p70, GM-CSF, and IL-10, exhibited a significant positive correlation with dsRNA levels in plasma. This cytokine signature indicates that dsRNA may serve as a trigger of systemic immune activation, potentially through engagement of PRRs such as RIG-I, MDA5, and TLR3. Nevertheless, we cannot exclude the possibility that an upstream immunological or viral factor independently drives both dsRNA accumulation and cytokine induction in parallel.

### 3.4 MS CSF is enriched in IP-10 and IL-8

**Figure 4:**
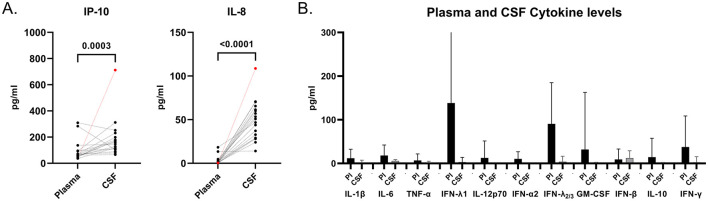
Elevated IP-10 and IL-8 levels in the CSF of MS patients. **(A)** Comparison of cytokine concentrations between matched plasma and CSF samples from treatment-naïve MS patients experiencing their first clinical visit (*n* = 18). IP-10 and IL-8 were significantly higher in CSF compared to plasma. Patient E cytokine concentrations are represented in red. **(B)** Distribution of additional measured cytokines in plasma (black) and CSF (gray) of MS patients (*n* = 18).

When cytokine concentrations were compared between CSF and plasma from treatment-naïve MS patients at their first clinical visit, IP-10 and IL-8 were found significantly elevated in the CSF (Figure 4A). The levels of the remaining cytokines are shown in Figure 4B. Notably, Patient E (indicated in red) exhibited the highest IP-10 and IL-8 concentrations among all CSF samples. This observation, together with the robust detection of dsRNA in the CSF of the same patient, suggests a potential link between intrathecal dsRNA accumulation and local ongoing viral activity or antiviral immune signaling within the CNS.

### 3.5 Atypical EBV reactivation in MS patients is associated with elevated plasma dsRNA and antiviral cytokine levels

As MS is closely linked to EBV infection, we next investigated EBV serology markers in the plasma and CSF of MS patients. Primary EBV infection (primoinfection), often resulting in infectious mononucleosis, is initially characterized by a rise in anti-VCA IgM, followed by the emergence of anti-VCA IgG and anti-EBNA1 IgG, which remain detectable throughout life and signify previous EBV exposure (Lupo et al., 2023). EBV can be reactivated upon external stress stimuli, such as another viral infection (Indari et al., 2024). EBV reactivation is frequently linked to elevated anti-EA(D) IgG, reflecting suspected EBV replication, and transient increases in anti-VCA IgM or IgA. Seropositive patients without symptoms of active infection are only positive for IgG antibodies. The concurrent detection of both anti-EBNA1 IgG and IgM antibodies is a rare finding and signals atypical EBV reactivation. Classification of MS patients into EBV serological groups was conducted using interpretation guidelines provided by the manufacturer (methods, Table 1), resulting in the following categories: EBV-seronegative (*n* = 0), primoinfection (*n* = 0), suspected EBV reactivation (*n* = 20; anti-EBNA1 IgG+ with anti-VCA IgA+, anti-VCA IgM+, or anti-EA(D) IgG+), seropositive without active EBV infection (*n* = 23; anti-VCA IgG+ and anti-EBNA1 IgG+), and atypical EBV reactivation (*n* = 8; anti-EBNA1 IgG+ and anti-EBNA1 IgM+) (Table 2).

**Table 2:** Demographic characteristics of MS patient subgroups and healthy controls.

**Sample**	**N**	**Age (m ± SD)**	**Sex (f/m)**
Total MS patients	70	36.9 ± 13.5	42/28
Seropositive	28	34.6 ± 16.4	17/11
Suspected reactivation	32	42.0 ± 16.1	16/16
Atypical reactivation	10	31.4 ± 5.1	7/3
Healthy controls	26	35.4 ± 13	18/8

Values represent number of subjects (N), mean age ± SD, and sex distribution (male/female).

**Figure 5:**
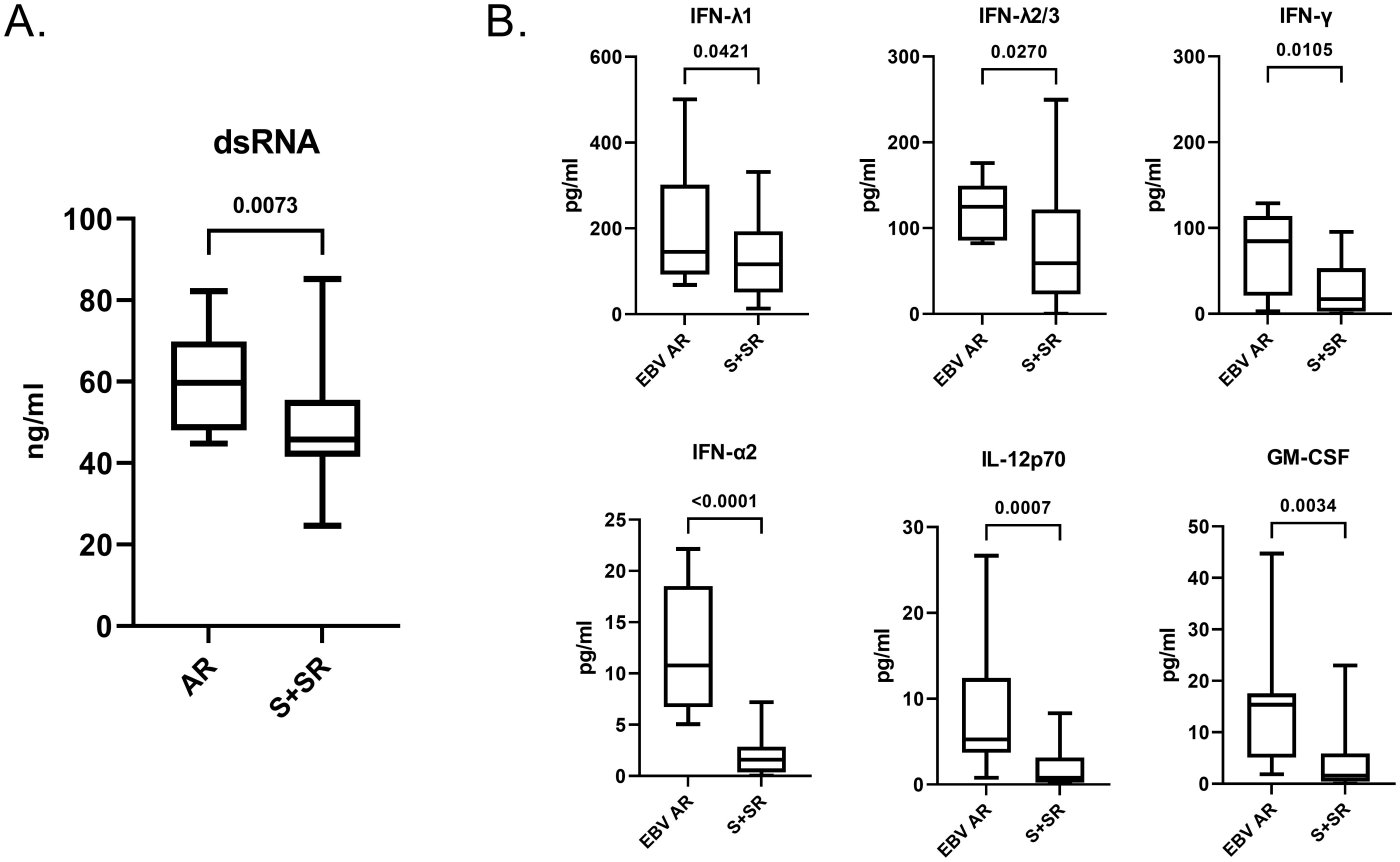
Plasma dsRNA and cytokine levels in MS patients. **(A)** Patients with EBV atypical reactivation (EBV AR, *n* = 10) have higher levels of dsRNA in the plasma compared to seropositive without active EBV infection (S, *n* = 28) and suspected EBV reactivation (SR, *n* = 32) grouped together (S + SR, *n* = 60). **(B)** Patients with atypical EBV reactivation (EBV-AR, *n* = 8) exhibited higher plasma levels of antiviral cytokines compared to S + SR (*n* = 43).

In our cohort, all MS patients showed previous infection by EBV, with no seronegative or primoinfection patients, consistent with previous reports (Abrahamyan et al., 2020). Patients with atypical EBV reactivation, showed higher dsRNA levels than all the other patients grouped together, which include seropositive patients without symptoms of active infection (S) and patients with suspected EBV reactivation (SR) (Figure 5A). Importantly, patients with atypical EBV reactivation showed significantly higher mean plasma levels of several antiviral cytokines, including IL-λ1 (Difference between means ± SEM, p-value: 80.88 ± 38.48, 0.0421), IL-λ2/3 (Difference between means ± SEM, *p*-value: 42.48 ± 25.24, 0.027), IFN-γ (Difference between means ± SEM, *p*-value: 40.60 ± 15.02, 0.0105), IFN-α2 (Difference between means ± SEM, *p*-value: 10.26 ± 1.061, < 0.0001), IL-2p70 (Difference between means ± SEM, *p*-value: 6.541 ± 1.457, 0.0007) and GM-CSF (Difference between means ± SEM, *p*-value: 12.11 ± 2.454, 0.0034) (Figure 5B), while seropositive patients without symptoms of active infection (S) showed no statistical difference compared to suspected EBV reactivation (SR) (data not shown).

### 3.6 MS patients exhibit impaired IgM-mediated humoral response to dsRNA

**Figure 6:**
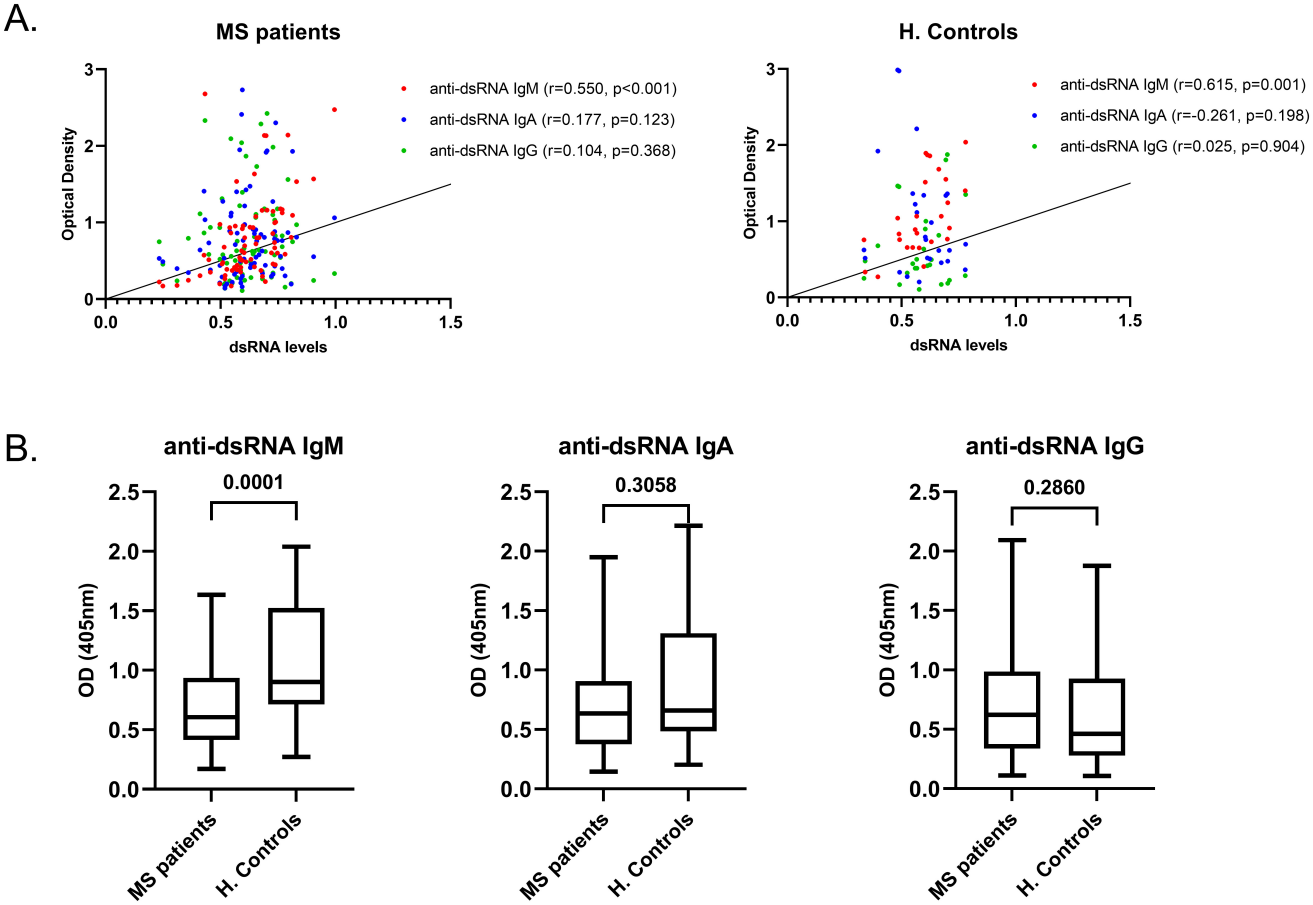
Correlation between dsRNA and anti-dsRNA antibody levels in plasma of MS patients and healthy controls. **(A)** Correlation between plasma dsRNA levels and IgM (red), IgA (blue), and IgG (green) anti-dsRNA antibody levels in MS patients (*n* = 70) and healthy controls (H. Controls, *n* = 26). **(B)** Anti-dsRNA reactivity measured for IgM, IgA and IgG antibodies in plasma of MS patients (*n* = 70) and healthy controls (H. Controls, *n* = 26).

An additional ELISA assay was developed to measure the levels of anti-dsRNA antibodies in the plasma of MS patients and to compare them with corresponding levels in healthy controls. Specifically, IgG, IgA and IgM antibodies targeting the dsRNA synthetic analog Poly(I:C) were measured in plasma samples from MS patients (*n* = 70) and healthy controls (*n* = 26), as well as in CSF samples from a subset of MS patients (*n* = 22). Among these, only anti-dsRNA IgM levels showed a positive correlation with plasma dsRNA concentrations in both MS patients and healthy controls (Figure 6A). Notably, MS patients exhibited significantly lower levels of anti-dsRNA IgM compared to healthy controls (Figure 6B). In addition, CSF was negative for IgM, IgG and IgA antibodies targeting dsRNA (*n* = 22). These findings suggest a potential impairment in the IgM-specific humoral response to dsRNA in MS, which may hinder effective clearance of immunostimulatory dsRNA structures following viral infection, thereby contributing to sustained inflammation in MS pathogenesis.

### 3.7 IgM anti-dsRNA antibodies correlate strongly with antiviral cytokine responses in MS patients

**Figure 7:**
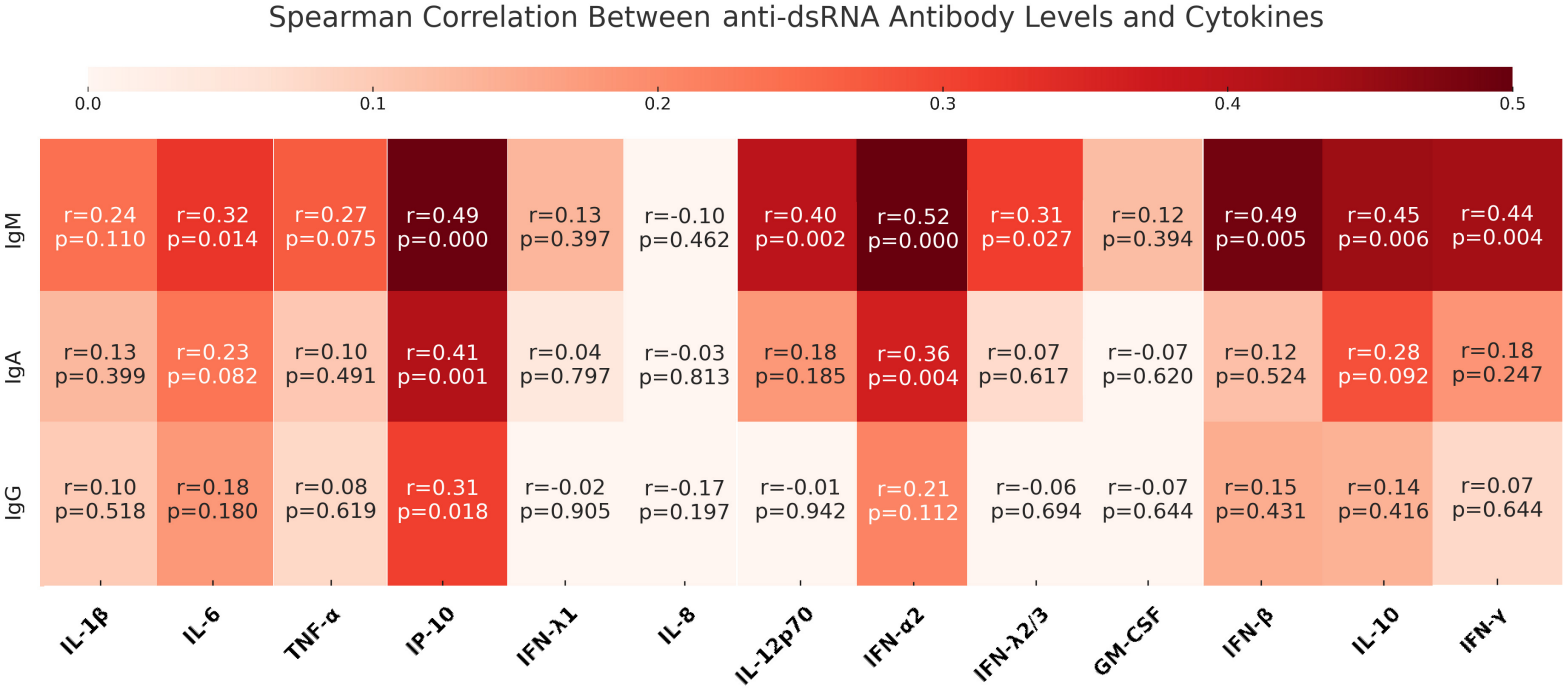
Heatmap for Spearman correlation between anti-dsRNA antibodies and antiviral cytokines in plasma of MS patients. Spearman *r* values and the corresponding *p* values are indicated and visualized in colored scales as shown in the top.

To further explore the relationship between the humoral response and antiviral immune activation, we assessed whether any anti-dsRNA antibody levels correlated with antiviral cytokine concentrations in the plasma of MS patients. We observed a positive correlation between anti-dsRNA antibodies and many of the antiviral cytokines measured (Figure 7). The strongest correlation was observed for IgM anti-dsRNA antibodies (IFN-λ2,3, IFN-α2, IL-6, IFN-γ, IL-12p70, IL-10, IFN-β and IP-10), while weaker correlations were observed for IgA (IFN-α2 and IP-10) and IgG isotypes (IP-10). The results indicate that in MS patients the link between anti-dsRNA antibodies and cytokine responses is largely driven by the acute-phase IgM response.

## 4 Discussion

In this study, we developed and optimized a sensitive and efficient sandwich ELISA for detecting dsRNA in human biological fluids. By leveraging the high affinity of two commercial J2 and K2 monoclonal antibodies for dsRNA structures longer than 40 bp, and incorporating Protein A precoating and the use of a F(ab')_2_ secondary antibody, the assay's signal-to-noise ratio was optimized compared to previously described protocols (Holland et al., 2024). We validated the increased specificity of the ELISA by using appropriate positive and negative reference samples. The assay robustly detected dsRNA in plasma and, to a much lesser extent, in CSF of MS patients, demonstrating its utility in clinically relevant samples.

We observed significantly elevated plasma dsRNA levels in therapy-naïve MS patients at their first clinical visit compared to healthy controls, supporting our hypothesis that dsRNA may act as an immunomodulatory signal in early MS pathogenesis. dsRNA is elevated in stress conditions such as viral infection and is recognized by PRRs, including RIG-I, MDA5, PKR, and TLR3, leading to the activation of the innate immune response and the production of antiviral cytokines (Chen and Hur, 2022). In line with this, we found that plasma dsRNA levels in MS patients showed a positive correlation with plasma levels of 13 antiviral cytokines. Notably, patients with atypical EBV reactivation (concurrent anti-EBNA1 IgG and IgM positivity) displayed both elevated dsRNA and cytokine levels (GM-CSF, IFN-γ, IFN-λ1/2/3, IFN-α2, and IL-12p70), suggesting that elevated dsRNA levels could be triggering secretion of antiviral cytokines during EBV reactivation in early MS. The role of EBV in other autoimmune and autoinflammatory diseases is robust (Borghol et al., 2025). To our knowledge, this is the first study to propose anti-EBNA1 IgM as a diagnostic marker and to introduce the term “atypical EBV reactivation” in the context of MS. Elevated plasma anti-EBNA1 IgM levels have also been reported in patients with rheumatoid arthritis (Trier et al., 2019).

While dsRNA was largely undetectable in the CSF, one patient with atypical EBV reactivation consistently exhibited relatively high CSF dsRNA levels across four independent experiments. The same patient was also positive for anti-EBNA1 antibodies in the CSF. EBNA1, a key EBV transcription factor, has been implicated in molecular mimicry with several CNS antigens, including GlialCAM, myelin basic protein, α-crystallin B, and Anoctamin-2 (Lanz et al., 2022; Cheng, 2012; Thomas et al., 2023; Tengvall et al., 2019). We cannot rule out the possibility of another concurrent viral infection, contributing to the elevated dsRNA and cytokine levels, which could also trigger atypical EBV reactivation. In addition, amongst the 13 antiviral cytokines tested, IL-8 and IP-10 were found significantly elevated in the CSF of MS patients compared to plasma. Previous reports have shown that dsRNA can induce the production of IL-8 and IP-10 (Voss et al., 2012; Taima et al., 2006). In addition, IP-10 has been found to induce lytic EBV reactivation through EXTL1 and IL-8 is a potent chemoattractant for EBV-infected B lymphocytes (Ding et al., 2024; Domínguez-Martínez et al., 2023). Interestingly, the patient with the highest dsRNA levels in the CSF, also exhibited among the highest levels of IP-10 and IL-8 in the CSF, which might indicate the activation of an antiviral response in the CNS. These findings align with prior research implicating EBV as a potential viral trigger of MS (Bjornevik et al., 2022) and raise the possibility that dsRNA detection may serve as a biomarker of active innate immune response or recent viral involvement in autoimmunity.

To investigate the adaptive arm of immune responses to viral RNA, we also developed a novel in-house indirect ELISA to detect antibodies against dsRNA (Poly(I:C)). Anti-dsRNA IgM antibodies showed a positive correlation with plasma dsRNA levels, whereas anti-dsRNA IgG and IgA did not, in both MS patients and healthy individuals. This suggests the existence of a mechanism whereby antibodies are generated not only against viral proteins but also against viral dsRNA itself. Interestingly, healthy controls had higher levels of anti-dsRNA IgM than MS patients. Thus, in contrast to antiviral cytokine production that is up-regulated, IgM anti-dsRNA antibody levels are down-regulated in early MS. A broad dysregulation of innate and early adaptive immune defense mechanisms would be consistent with a growing body of evidence that MS may involve an inability to mount an effective acute-phase antiviral humoral response, which might result in failed containment of latent viral infections, ultimately triggering autoimmunity through molecular mimicry (Bjornevik et al., 2022; Lanz et al., 2022).

In conclusion, we report the development of a sensitive and specific sandwich ELISA capable of detecting nanogram quantities of dsRNA in human biological samples. This method revealed elevated plasma dsRNA levels in early MS, with dsRNA levels positively correlating with antiviral cytokines, particularly in patients with serological evidence of atypical EBV reactivation (anti-EBNA1 IgG^+^ and anti-EBNA1 IgM^+^). The ability of this assay to detect dsRNA in plasma and CSF highlights its diagnostic and mechanistic value as a general non-invasive biomarker of endogenously or exogenously driven inflammation in biological fluids. Moreover, the assay's adaptability and robustness make it a promising tool for broader applications beyond MS, including other autoimmune or post-viral syndromes, such as SLE, viral encephalitis, and long COVID. Importantly, the concurrent detection of anti-dsRNA antibodies suggests a novel “arm” of the immune system that may actively recognize and respond to immunomodulatory dsRNAs, potentially as a means of re-establishing homeostasis following viral infections. Together, these findings open new avenues for exploring the role of dsRNA in neuroinflammation and immune dysregulation and may contribute to the advancement of precision diagnostics, personalized monitoring, and targeted antiviral therapies in MS and related disorders.

## Data Availability

The raw data supporting the conclusions of this article will be made available by the authors, without undue reservation.
